# Exploring Early Stages of the Chemical Unfolding of Proteins at the Proteome Scale

**DOI:** 10.1371/journal.pcbi.1003393

**Published:** 2013-12-12

**Authors:** Michela Candotti, Alberto Pérez, Carles Ferrer-Costa, Manuel Rueda, Tim Meyer, Josep Lluís Gelpí, Modesto Orozco

**Affiliations:** 1 Institute for Research in Biomedicine (IRB Barcelona), Barcelona, Spain; 2 Joint Research Program in Computational Biology, Institute for Research in Biomedicine and Barcelona Supercomputing Center, Barcelona, Spain; 3 Laufer Center, Stony Brook University, Stony Brook, New York, United States of America; 4 Skaggs School of Pharmacy and Pharmaceutical Sciences, University of California San Diego, La Jolla, California, United States of America; 5 Theoretische und Computergestützte Biophysik, Max-Planck-Institut für Biophysikalische Chemie, Göttingen, Germany; 6 Department of Biochemistry and Molecular Biology, University of Barcelona, Barcelona, Spain; University of Uppsala, Sweden

## Abstract

After decades of using urea as denaturant, the kinetic role of this molecule in the unfolding process is still undefined: does urea actively induce protein unfolding or passively stabilize the unfolded state? By analyzing a set of 30 proteins (representative of all native folds) through extensive molecular dynamics simulations in denaturant (using a range of force-fields), we derived robust rules for urea unfolding that are valid at the proteome level. Irrespective of the protein fold, presence or absence of disulphide bridges, and secondary structure composition, urea concentrates in the first solvation shell of quasi-native proteins, but with a density lower than that of the fully unfolded state. The presence of urea does not alter the spontaneous vibration pattern of proteins. In fact, it reduces the magnitude of such vibrations, leading to a counterintuitive slow down of the atomic-motions that opposes unfolding. Urea stickiness and slow diffusion is, however, crucial for unfolding. Long residence urea molecules placed around the hydrophobic core are crucial to stabilize partially open structures generated by thermal fluctuations. Our simulations indicate that although urea does not favor the formation of partially open microstates, it is not a mere spectator of unfolding that simply displaces to the right of the folded←→unfolded equilibrium. On the contrary, urea actively favors unfolding: it selects and stabilizes partially unfolded microstates, slowly driving the protein conformational ensemble far from the native one and also from the conformations sampled during thermal unfolding.

## Author Summary

The delicate equilibrium between the folded and functional structure of a protein and its unfolded state is highly dependent on environmental variables such as the solvent. For example the co-solvent urea is a well-known protein denaturant that displaces the equilibrium towards unstructured and non-functional conformations of proteins. However the molecular mechanism behind its ability remains an enigma and the interpretation of the experimental data is still ambiguous. By analyzing a set of representative proteins through extensive molecular dynamics simulations in urea, we provide a robust and consensus picture of the first stages of urea-driven protein unfolding and elucidate the role of urea in accelerating protein unfolding. Our results suggest that urea, thanks to its stickiness and slow diffusion, benefits from the intrinsic flexibility of proteins and stabilizes partially open-states, slowly driving the protein toward unfolding.

## Introduction

Urea is a protein denaturant that has been used for decades in the study of protein folding/unfolding; however, after many years of research the ultimate reasons of the denaturing properties of urea remain elusive [1], [2]. The dominant paradigm for unfolding (the “direct” mechanism) claims that the denaturant properties of urea are related to its capacity to interact with exposed protein residues more strongly than water [3]–[15]. However, the nature of such a preferential interaction is not so clear. Thus, while some authors suggest that it is mostly electrostatic and related to the formation of direct hydrogen bonds [7]–[9], [16]–[17], others claim that preferential dispersion is the leading term [13]–[15]. It is also unclear whether the major destabilizing effect of urea is related to interaction with the backbone [6]–[7] or with side chains [8]–[12]. In the latter case, there is also discussion regarding the preferential side chains: polar and charged [9] or apolar [4], [10]–[12].

We recently combined multi-replica molecular dynamics (MD) simulations and direct NMR measures of ubiquitin to characterize the “urea unfolded ensemble” of this model protein [15]. Our results suggest that urea stabilizes flexible over-extended conformations of the protein, which are unlikely to be sampled in the “unfolded” state of aqueous proteins. Extended conformations of the protein with exposed hydrophobic surfaces are more urea-philic than the native globular state, due mostly to extensive London dispersion interactions (the attractive contribution in Van der Waals interactions between instantaneous dipoles) between apolar side chains and urea molecules in the first solvation shell of unfolded conformations. We believe that our results in reference 15 clarify the molecular basis of the effect of urea on the thermodynamics of the folded←→unfolded equilibrium, but unfortunately, they do not provide information on the kinetic role of urea in the unfolding process. In other words: does urea actively induce protein unfolding? Or, on the contrary, does it passively stabilize the unfolded state by selectively binding to unfolded conformations? To analyze this point, we should characterize the effect of urea in the first stages of thermochemical unfolding, when the protein structure is still close to the native conformation and internal residues are not fully exposed. Clearly, a study of this nature presents many difficulties, the most important being that the effect of urea on early stages of unfolding might be dependent on the native structure. Therefore, to obtain conclusions of general validity, all representative protein folds should be addressed. Also, results can be force-field-dependent, so if we aim to obtain robust conclusions, we should perform simulations with a variety of force-fields.

Given the typical kinetics of the folding/unfolding transitions of small globular proteins [18], microsecond (µsec) long simulations should trace the first stages of these processes. In the current work, we investigate the first stages of urea-driven protein unfolding using µsec-long atomistic simulation; to gain universality, we used 30 proteins representative of all protein folds, while to protect our conclusions from force-field-related uncertainties, we used several of the most popular force-fields. The results derived from this study provide a robust and complete picture of the role of urea in destabilizing folded states of proteins, and more importantly, on the molecular mechanisms by means of which urea contributes to accelerating protein unfolding.

## Results

### Protocol validation using three ultra-representative proteins

We first validated our protocol using three ultra-representative proteins (in bold in Table 1), one for each of the main classes in the Structural Classification of Proteins (SCOP, [19]). We monitored the protein stability in three environments: i) in chemical unfolding conditions, in 8M urea and with a mildly high temperature (T = 368K) to speed up the observable effects; ii) in thermal unfolding condition, in water with the same high temperature; this control allowed us to distinguish the effect of urea and temperature on protein unfolding; iii) in water at room temperature as final control. Four force-fields were used (OPLSAA - ON2; CHARMM - C22; AMBER99 - P99 and P99SBILDN) for each system (see Methods for the description of the force-fields used), collecting in total 36 simulations of 1-µsec length each.

**Table 1:** Structures representative of the 30 most populated protein meta-folds.

Symb Fig. 3	PDB code	Molecule name
a	1AGI	Angiogenin-1
b	1CHN	Chemotaxis protein CheY
c	1FVQ	Copper-transporting ATPase
d	1GND	Rab GDP dissociation inhibitor alpha
**e**	**1KTE**	**Glutaredoxin-1 (Thioltransferase)**
f	1LIT	Lithostathine-1-alpha
g	1PDO	Mannose Permease – IIA domain
h	1SDF	Stromal cell-derived factor-1
i	1SUR	PAPS Reductase
j	2HVM	Hevamine
k	1BFG	Basic fibroblast growth factor
l	1BJ7	Allergen Bos D2
**m**	**1CQY**	**β-amylase, Starch-binding domain**
n	1CSP	Cold shock protein B
o	1CZT	Coagulation factor V, C2 domain
p	1J5D	Plastocyanin
q	1KXA	Sindbis virus capsid protein
r	1NSO	Protease
s	1PHT	P13-kinase, SH3 domain
t	1BSN	F1-ATPase, ε subunit
u	1EMR	Leukemia Inhibitory Factor
v	1IL6	Interleukin-6
w	1JLI	Interleukin-3
x	1K40	Focal adhesion kinase, FAT domain
y	1LKI	Leukemia Inhibitory Factor
z	1OOI	Odorant binding protein LUSH
**α**	**1OPC**	**OMPR, Dna-binding domain**
β	1FAS	Fasciculin-1
γ	1I6F	Alpha-like toxin CsEv5
δ	1SP2	SP1F2, zinc-finger dna binding domain

*all-α*, *all-β and α/β*). The three ultra representative proteins used in the protocol validation are in bold. The list is divided according to the SCOP fold group (in order

#### Control simulations at room temperature

**Figure 1:**
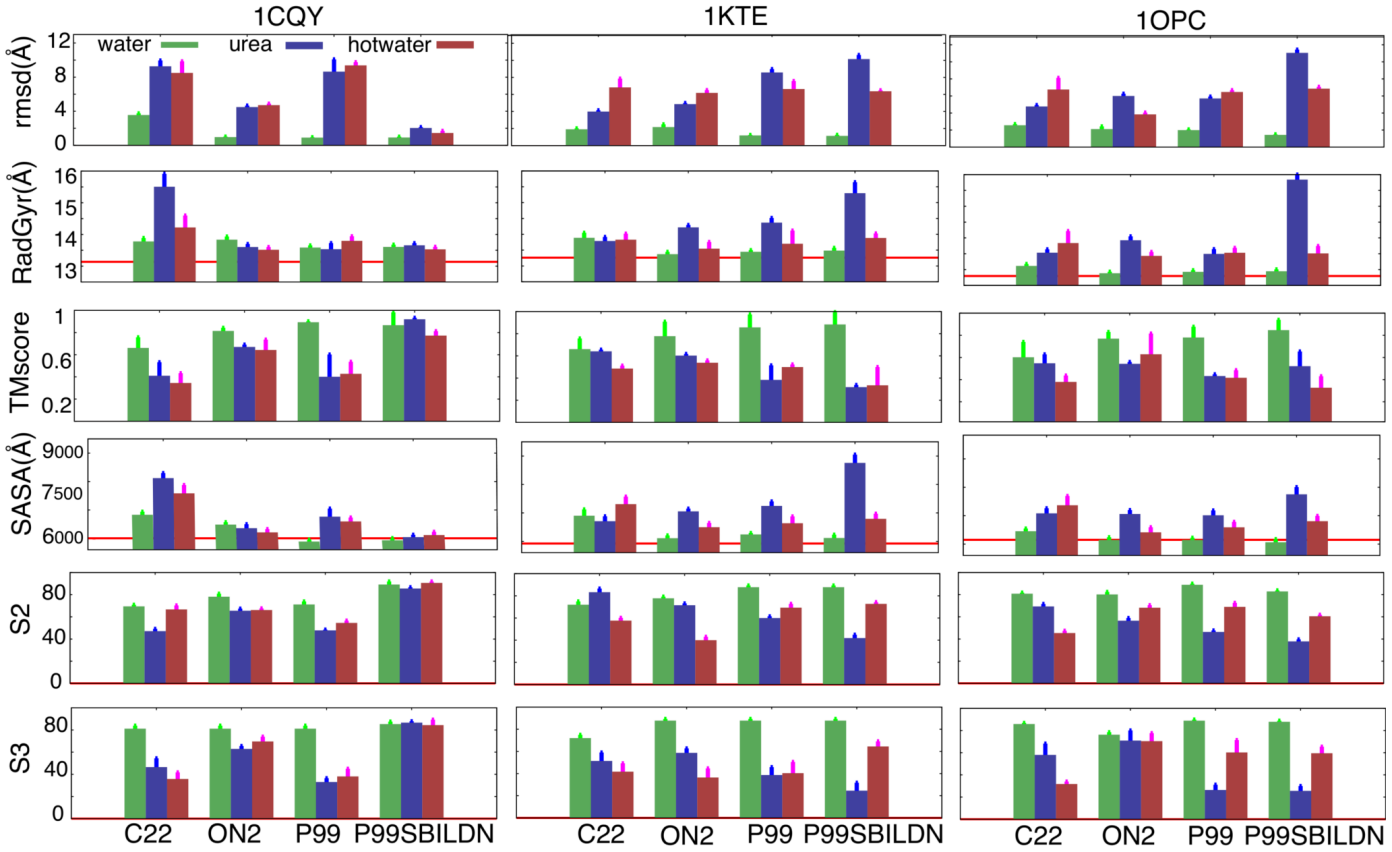
Shape and unfolding descriptors for the three ultra-representative proteins. Root-mean-squared-deviation (RMSD) from the starting conformation, radius of gyration (RadGyr), TMscore, solvent accessible surface area (SASA), native secondary structure index (S2) and native contacts index (S3) were calculated in water, urea and hot water in the four force-fields (OPLSAA - ON2; CHARMM - C22; AMBER99 - P99 and last-modified P99SBILDN). Average values and relative standard deviations are calculated in the last 10 ns of the simulation. For radius of gyration and SASA, the value found for the starting conformation is reported as a red line. See Methods and Suppl. Text S1 for a description of the metrics. Error bars mark the standard deviation.

Analysis of the trajectories in water at room temperature for the 3 ultra-representative proteins confirmed that current force-fields can accurately represent the native conformation of soluble proteins in the µsec range [20], [21], [22], reproducing the global and local structure of proteins well. The structures in the last segment of the trajectory (and the corresponding ones collected just after equilibration) showed, in general, little structural drift from the experimental conformation (see Figure 1, Suppl. Figures S1, S2 and Suppl. Table S1). This was noted in the small values of root mean squared deviation (RMSD) from native structure at the end of the simulation (typically around 1.5 Å), and the good preservation of the fold structure (average TMscore around 0.8), the shape descriptors (radius of gyration, RadGyr and solvent accessible surface area, SASA) and the secondary structure (SS) composition. We found only one significant discrepancy: simulation of 1CQY using the C22 force-field showed a non-negligible transition in the 100-ns time scale, leading to the sampling of conformations that were 3 Å away from the experimental structure; see Suppl. Figures S1, S2.

#### Control simulation of thermal unfolding (hot water)

The mild high temperature applied in the simulations in hot water (below water boiling point: T = 368K) significantly enhanced the global fluctuations of the protein (see Suppl. Figures S2), while advances in the unfolding were still moderate. Thus, after 1 µsec of MD in hot water, the RMSD from experimental structures reached the range 5–7 Å, and shape descriptors (RadGyr and SASA) indicated a moderate increase in the size of the protein (see Figure 1 and Suppl. Table S2). The fold-architecture started to be corrupted (TMscore values around 0.5), with a moderate loss of native contacts (S3) and native secondary structure (S2 - see Figure 1 and Suppl. Table S3).

Detailed analysis of the 12 simulations (three proteins and four force-fields) provides interesting information on the behavior of the force-fields. In general, the overall picture at the beginning of thermal denaturation of proteins was quite robust to force-field changes. However, we found two clear discrepancies. First, C22 appeared to facilitate unfolding in hot water (see Suppl. Figure S2), yielding more flexible structures than those obtained with the other force-fields. Second, in P99SBILDN the 1CQY protein remained fully preserved at the end of the high temperature trajectory. Five independent replicas of the same system with different starting geometries and velocities failed to detect significant unfolding for 1CQY with P99SBILDN. This observation points to a potential problem of over-stabilization of the folded structure for this all-β protein.

#### The differential effect of urea in the early stages of chemical unfolding

We first analyzed the impact of high concentrations of urea on the three ultra-representative proteins. Overall, and contrary to previous suggestions [13], in the microsecond scale the unfolding efficiency of urea did not change dramatically from that in hot water simulation. In the same simulation period, proteins in urea display RMSD values that were marginally larger than in hot water (see Figure 1 and Supp. Table S2), and TMscore, S2 (secondary structure) and S3 (native contacts) values at the end of the simulations in urea were not much different to those obtained in hot water (see Figure 1 and Supp. Table S2), except for a certain enlargement in the disruption of β-sheets when urea was added (see Suppl. Table S3).

**Figure 2:**
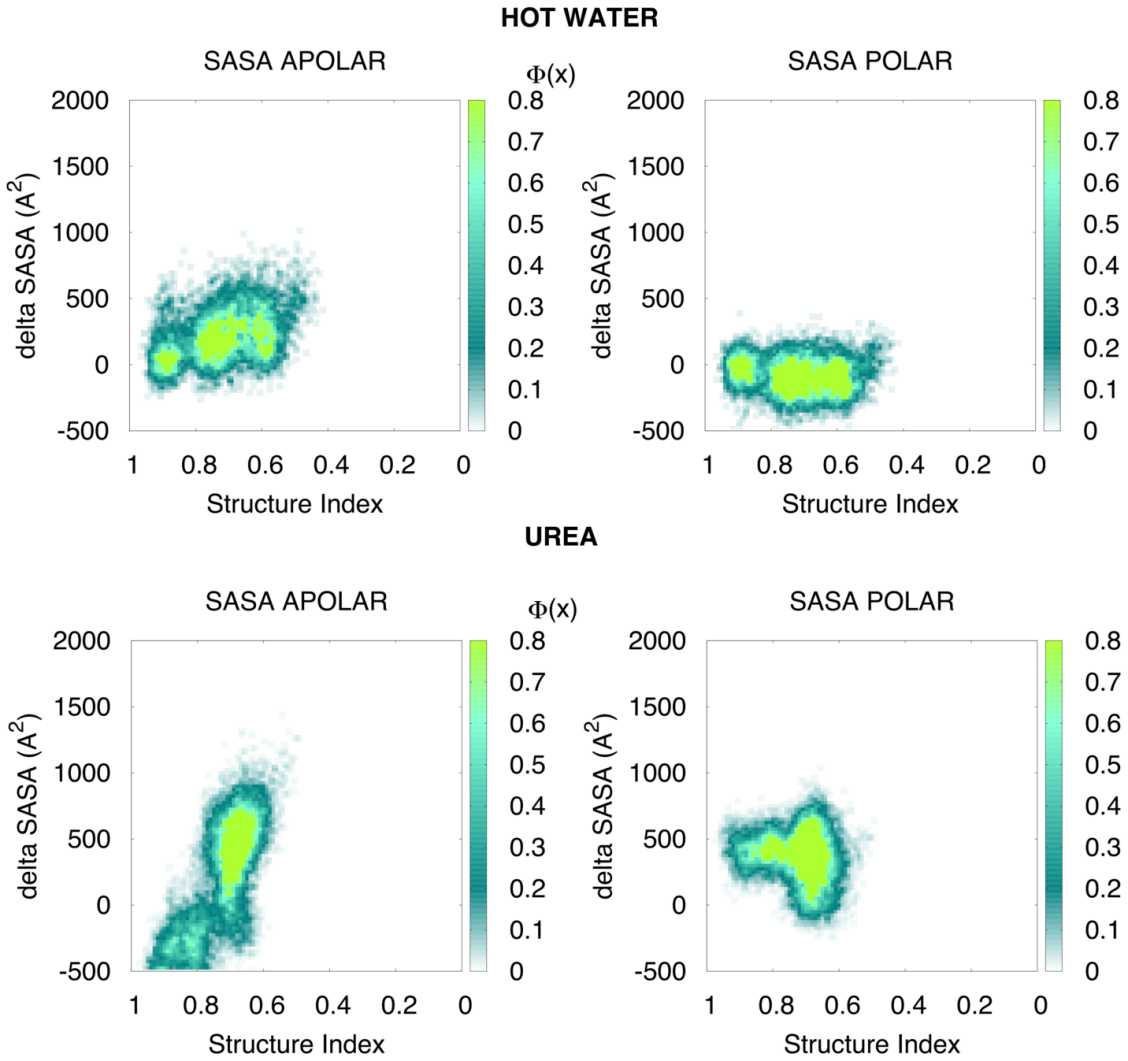
Variation of the solvent accessible surface area (SASA) during the unfolding. Values are reported for apolar and polar residues in hot water and urea, for the three ultra-representative proteins in all force-fields. SASA is normalized for each protein using the average value calculated in the water simulations (to take into account the structure rearrangements and the mobility in water), while the structure index is used to follow the unfolding process (from 1 - fully native folded protein - towards 0). The color marks the density. For more detailed pictures for each protein see Suppl. Figure S3. Note that in urea the increase in SASA is larger than that in water, mostly due to the exposure of apolar moieties, similarly to the action of surfactant.

We found a significant correlation (r = 0.701; p-value<2.2 10^−16^) between the time that each native contact remained lost in water and in urea at the same temperature (Suppl. Figure S3-A). This observation suggests that urea does not attack specific parts of the protein, but rather benefits from the intrinsic breathing movements of the protein at high temperature. However, the role of urea in guiding unfolding is reflected by the different nature of the structural deformations that occurred in hot water and urea simulations. Thus, the latter sampled conformations that were slightly more extended (higher RadGyr) and clearly more exposed (higher SASA) than those sampled in hot water (Figure 2). It is worth noting (see Figure 2 and Suppl. Figure S3) that in urea-driven unfolding the solvent-accessible surface (SASA) corresponding to apolar residues increased dramatically, a behavior reminiscent of the surfactant action, while this increase was moderate in hot water simulations. The urea-induced increase of the apolar-exposed area was not accompanied by a dramatic enlargement of RadGyr or to a large decrease in the structural indexes, thereby suggesting that the exposure of the hydrophobic core occurs through the creation of small cavities (filled with urea) and the exposure of apolar side chains, without a dramatic extension of the protein or an explosion of the hydrophobic core.

A second major difference between the unfolding yielded by hot water and by urea was revealed by the analysis of the dynamics of the protein. Intuition suggests that proteins will show greater fluctuation (at the same temperature) in the presence of a denaturant like urea. This is certainly true for a fully unfolded protein [15], but not during early stages of unfolding, when the protein is still close to its native state, as noted in the values of RMSD calculated in various time-windows (see Suppl. Figure S3-B). The explanation of this apparently counterintuitive finding is that urea solutions are more viscous, thus reducing the fastest movements of the proteins, including the oscillations of side chains (66% of side chains were stiffer in urea than in water). This reduction causes a slow down of the atomic-motions, which in fact opposes unfolding. However, the slower mobility of urea and its sticky nature may explain the longer life time of lost contacts (see Suppl. Text S1) in urea (see Table 2 and Suppl. Figure S4-C), a feature that clearly favors unfolding (see below).

**Table 2:** Change in flexibility of contacts (opening time) maintained in hot water and urea.

Force-field	U* (%)	W* (%)	Tot (%)	Δ(U-W) Normalized (%)
**ON2**	10.54	7.4	17.94	+17.5%
**P99**	12.19	4.64	16.83	+44.8%
**C22**	2.76	5.85	8.61	−35.8%
**P99SBILDN**	7.02	3.51	10.53	+33.3%

∼380 native contacts - defined as those occurring for more than 80% of the time in the 0.1 microsecond simulation in water at 300K. Percentage of native contacts that present a longer opening time in urea (U) or water (W) (difference between opening time larger than | 0.1 | ns). The average total number of contacts is 1110 in C22, 1148 in ON2, 1140 in P99 and 1143 in P99. Each protein has

Regarding differences related to force-fields, we detected the same discrepancies as in hot water. C22 simulations showed more mobility and distortions (Suppl. Figure S2), but conformations were still similar to those obtained with other force-fields. With the P99SBILDN force-field, the full-β protein 1CQY remained stable when simulated at high temperature in the presence (but also absence) of urea. Five 1-µsec replicas of this trajectory failed again to detect any significant unfolding of this protein, a finding that suggests caution in the use of P99SBILDN (a force-field refined to reproduce folded structures) in unfolding studies of full-β proteins. Given our observation, the P99SBILDN force-field was not considered in the rest of the study.

### Proteome-level study of urea unfolding

After the validation of our protocol, we extended the chemical unfolding simulations to a larger set of proteins, to avoid any bias in the conclusion due to the native structure. We performed 1 µsec of simulation in urea at high temperature (T = 398K) for 30 proteins covering all the major protein folds (Table 1 and Suppl. Dataset S1). Each system was simulated in three force-fields (C22, ON and P99), excluding P99SBILDN as reported above, and collecting in total 90 simulations. To have a more realistic picture of the native state, instead of using the crystal structure, we used as control 0.1 µsec-long simulations in water at room temperature for all the 90 systems. The analysis described here reveals some common robust trends that illustrate the effect of urea during the early stages of protein unfolding.

#### Global denaturation

**Figure 3:**
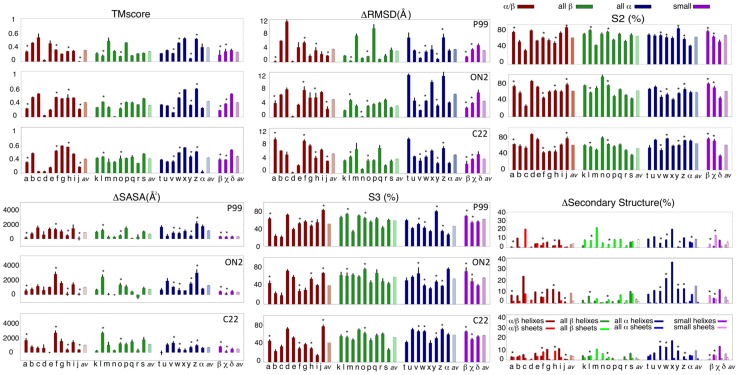
Shape and unfolding descriptors for the 30 representative proteins. The difference of TMscore, RMSD and SASA between values in urea and in water (to allow comparison between proteins of different size) calculated in urea in the three force-fields. The native contacts index (S3), native secondary structure index (S2) and the difference in Secondary Structure content (Δ% Sec. Structure) are also shown. To facilitate discussion, proteins are grouped following the SCOP classification. The correspondent pdb code is reported in Table 1; the group average is reported as “*av*” while the symbol * marks proteins with disulfide bonds. Error bars mark the standard deviation.

As anticipated from simulations in the small set of ultra-representative proteins, urea led to an enlargement of the protein and to a deviation from its native structure (Figure 3), without reaching, however, full unfolding in any of the 90 simulations in urea at high temperature. On average, our simulations produced RMSD values (from experimental native conformation) around 4 Å larger than those found at the end of the control simulation in water, while for these proteins a fully unfolded structure should yield ΔRMSD values above the range 20 Å [15] and a random structure above 10 Å [23]. Only a few proteins lost their fold integrity and native contacts after 1-µsec simulations in urea at high temperature, as noted in the reductions beyond 0.5 in the TMscore and in native contact (S3 structural index) around 0.2–0.3. However, in general, the urea-induced disruption of core structural elements was moderate (reduction of TMscore around 0.3–0.4 and S3 indexes around 0.4–0.6 at the end of the trajectory; see Figure 3).

The selected force-fields showed a consistent representation of unfolding and in general the urea- labile or resistant proteins defined among the entire set matched in all of them. For example, all force-fields detected minuscule advances in unfolding (as determined by the set of metrics in Figure 3) for 1GND(d), 2HVM(j), 1CSP(n), 1OPC(α) and 1KTE(e), whereas the same force-fields detected significant progresses in others (for example 1CQY-m, 1BSN-t, 1OOI-z, 1K40-x or 1SP2-δ). Only in a few cases was there apparent large discrepancy between force-fields (example 1FVQ) and these corresponded to simulations where structural alterations were already seen in the reference simulations (example C22 for 1FVQ). In summary, despite the stochastic nature of unfolding and the uncertainties implicit to the force-field, the general picture of urea unfolding detected here is robust.

#### Urea-induced unfolding and loss of secondary structure

We did not find any correlation between the presence/absence of disulphide bridges and the extent of urea-induced unfolding (note that to exclusively analyze the effects of urea, disulphide bridges were not reduced in our calculations). All changes in urea sensitivity related to fold type, secondary structure composition or the presence or absence of disulphide bridges were small during the first stages of unfolding.

#### The distribution of urea around the protein

**Figure 4:**
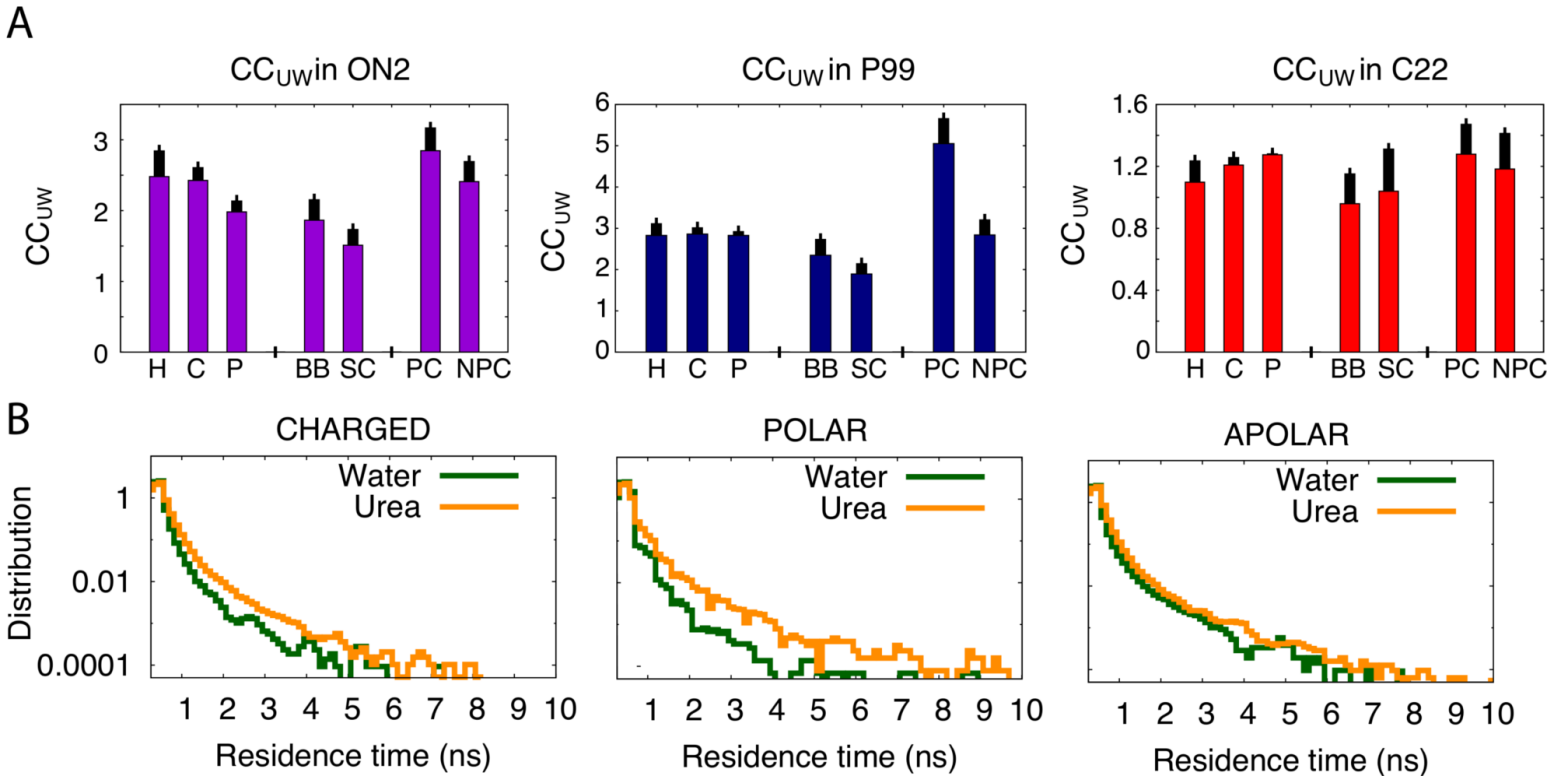
Location of solvent molecules in urea. **A**) Preference for urea solvation measured by CC_UW_ (the ratio for each amino acid between atomic contacts with urea and with water molecules - see Suppl. Text S1) for different parts of the protein: hydrophobic (H), polar (P), charged (C) residues, side chains (SC), backbone (BB), protein core (PC) and non-protein core (NPC). Error bars mark the standard deviation. Note that in all the force-fields the PC shows the largest values, meaning a larger preference for urea. **B**) Distribution of the residence time for urea and water molecules during a 1-µsec trajectory.

As anticipated in previous studies [4], [8], [10], [13], [15], proteins are urea-philic. All the proteins studied here (for all force-fields) quickly recruited urea into the first solvation shell (in agreement with osmometric experiments [24]), where the water/urea ratio reached values in the range 3–3.5 water/urea molecules, while the background ratio was around 6 (see Table 3). However, this enrichment was smaller than that found for a fully unfolded protein (0.9 for unfolded ubiquitin; [15]), thereby suggesting that the most urea-philic groups remained buried in the interior of the protein. Urea did not preferentially solvate any residue (see Figure 4A) and showed preferential binding to the backbone rather than to the side chains during the first stages of unfolding. Although larger in size, urea has a higher affinity than water to interact with residues placed in narrow cavities near the hydrophobic core (see below). Long residence urea molecules placed in these cavities led to a partial exposure of the hydrophobic core of the protein (see Figures 1–3).

**Table 3:** Comparison of ratio water-urea in the first solvation shell (FSS) and in the bulk (BULK).

Ratio_WU_ *	ON2FSS^1^ - BULK^2^	P99FSS - BULK	C22FSS - BULK
**All α**	3.39–6.26	3.01–6.45	5.03–5.87
**All β**	3.56–6.43	3.13–6.52	5.12–5.89
**α/β**	3.22–6.24	3.09–6.21	4.93–5.94
**Small**	3.46–6.17	2.94–6.33	4.95–6.19

Values are the average along the simulation, SD is always lower than 0.2.

values are the average along the simulation, standard deviation is always lower than 0.2,

Å cutoff to protein,^1^ FSS defined by a maximum 5

Å.^2^ BULK defined by a minimum cutoff of 6

At this point we wish to comment on the urea distribution for C22 simulations, since we detected significantly less urea in proximity of the protein as compared to the other force-fields. This unusual behavior of C22 urea simulations is evident in Table 3 and Suppl. Figure S5-AB, where some trends found in P99, ON2 (or P99SBILDN) differ from those in the C22 simulations. Urea densities around the proteins in the C22 simulations may have been too low, possibly reflecting the excessive polarity of the urea model used in the C22 trajectories (dipole moment 5.3 D, compared with the dipoles around 4.7 D of the other models) [11].

#### The energetics of protein-urea interaction

The nature of the interaction between urea and proteins has been the subject of intense discussion (see *Introduction*). Our previous results [15] suggest that in the fully unfolded state there are many urea-protein hydrogen bonds, mostly with the backbone, but that the main factor responsible for the urea-philicity shown by proteins is the differential dispersion interaction of bulk and protein-bound urea. However, these conclusions for the unfolded state might not be valid when the protein is still compact during the early stages of unfolding. Analysis of current data (see Table 4 and Suppl. Table S4) shows that already in these early stages of unfolding 30% of the protein-solvent hydrogen bonds are with urea, and the ratio is even higher (36%) when considering only stable contacts. In 2/3 of the cases, urea acts as a hydrogen donor when H-bonding to the backbone, and, in general, urea-protein H-bonds display longer life times than water-protein ones, a feature that appears to be crucial to stabilize partially exposed residues (see below). Nevertheless, the formation of these H-bond interactions (mostly electrostatic in nature) is not the driving force that explains the urea-philicity of the nearly-native conformation, since the migration of urea from background to the first solvation shell (FSS) of the protein does not alter global electrostatics (see Suppl. Figure S5-B), but improves the van der Waals interactions [13]–[15]. This effect and the gain in water entropy related to the replacement of several water molecules by a single urea molecule [10], [11] may drive denaturation in the early stages of urea unfolding.

**Table 4:** Hydrogen bond interactions of urea/water with proteins during the last 10 ns of trajectories.

H- bonds (% of total)*:	Urea/water as H-donor	Urea/water as H-acceptor
	66/65	34/35

H-bonds with protein:	Backbone	Side chains	Backbone	Side chains
% of total	58/48	42/52	67/69	33/31
Av. Lifetime(ps)$	74/64	107/74	327/68	451/249

Å, angle cutoff is 120.00 degrees. Hydrogen bonded solvent molecules are defined for occupancies (total time) larger than 0.5 ns, Distance cutoff is 3.50

Life-time refers to the percentage of analyzed trajectory (10 ns).

#### Urea and protein dynamics

**Figure 5:**
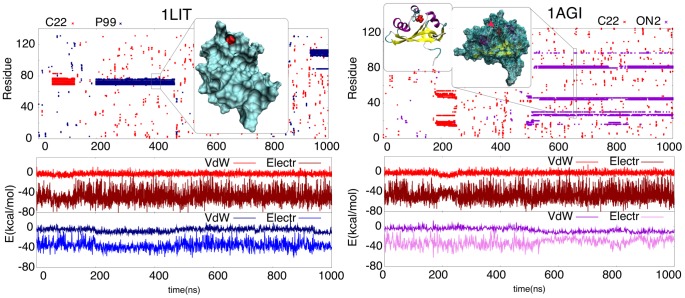
Long residence urea molecules. Examples of urea contacts with the protein residues (y-axis) along 1 µsec of simulation (x-axis). Each dot defines a contact between a urea molecule and protein residues. A contact is defined when at least one pair of heavy atoms comes closer than 3.5 Å (see Suppl. Text S1). Examples of urea molecules trapped in the protein core are shown in the top panels. Note that in the same protein but simulated in different force-fields, a long residence urea is trapped in a very similar area of the protein core. The panels below show the evolution of electrostatic and dispersion energies for the urea molecules (calculation details as in Suppl. Figure S5-B; see also Suppl. Text S1). Note the reduction mainly in dispersion energies upon the binding of urea.

Urea diffuses quite slowly (Suppl. Figure S5-C) and limits protein fluctuations, which leads to an apparent paradox: a denaturant that slows down the dynamics of proteins compared to the equivalent simulations in water (see also Suppl. Figure S4-B). However, analysis of trajectories show that such a paradox does not exist. Urea migration to the protein surface was slower than that of water, but once it reached the surface, urea remained for longer periods (see Figure 4B), especially when located in cavities near the hydrophobic core of the protein (see Figure 5 and Suppl. Figure S6-A for examples). Interestingly, the positions of long-lasting urea interactions are consistent among all four force-fields and seems associated with a sizeable improvement in van der Waals interactions and electrostatic energies and with the formation of strong long-living H-bonds (see examples in Figure 5, Suppl. Figure S6-A and Suppl. Table S4). These findings demonstrate that even if H-bonding is not the driving force behind the urea-philicity of proteins, it is important to stabilize urea molecules at specific positions at the protein interior.

**Figure 6:**
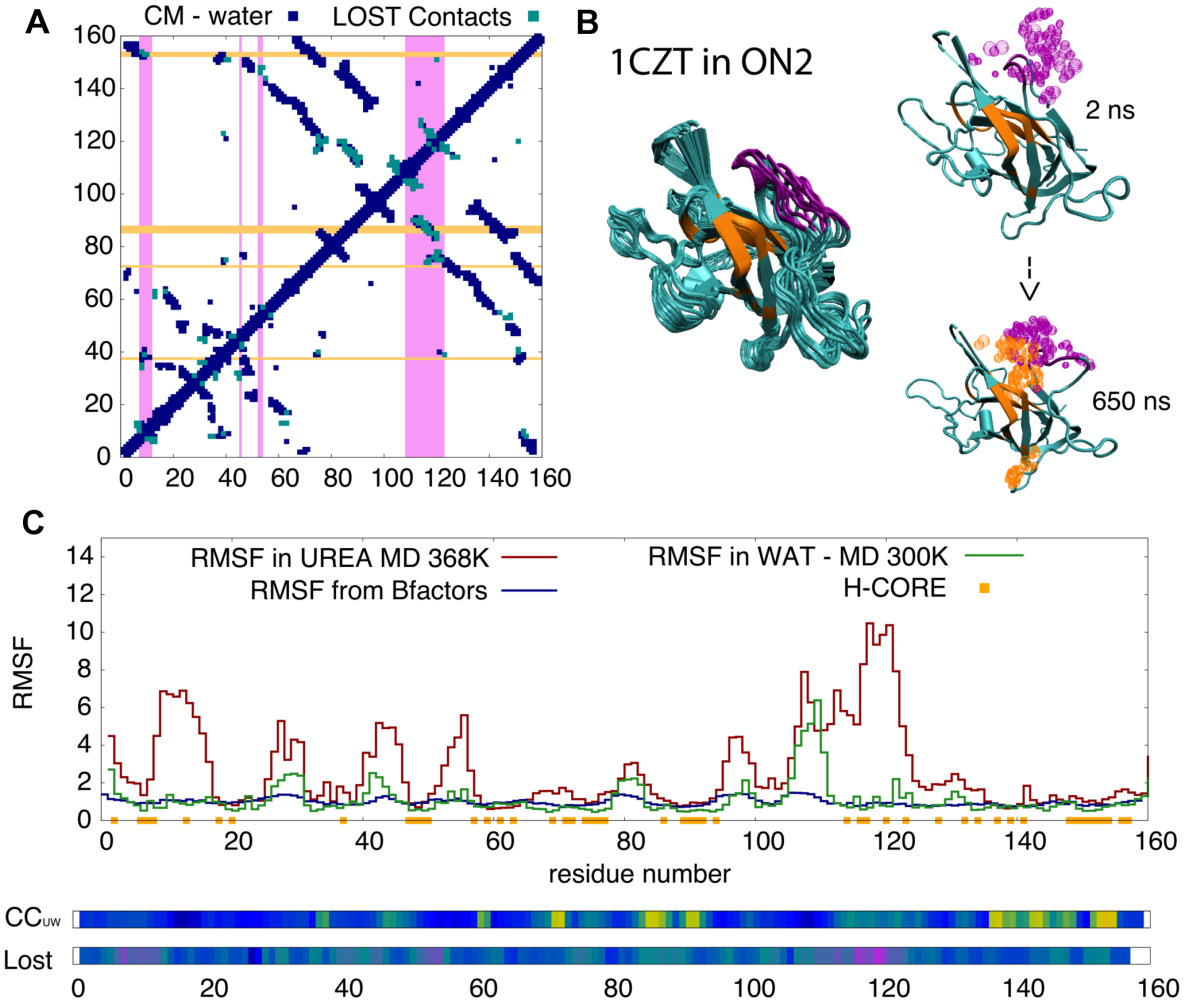
Urea intrusion into the core of 1CZT. **A**) Contact map from the crystal structure of 1CZT in blue (each dot represent a contact), the contacts lost during 1 µsec of simulation in urea are shown in light blue. Areas in magenta mark residues with a large flexibility while those in orange mark residues with a high preference to contact urea. **B**) Snapshots showing the temporal evolution of the protein structure; the areas in magenta and orange follow the same color code as in panel A. Urea molecules within 4 Å of these areas are shown in the same color. Note that flexible areas (in magenta) on the surface of the protein - mainly loops - undergo opening events, and the loss of contacts (panel A) connecting these areas to the protein core (in orange) triggers urea intrusion. **C**) The residue root mean square fluctuation (RMSF; a measure of flexibility), the contact coefficient CC_UW_ (measure of the binding preference of protein to contact urea rather than water) and the % of lost time (measure of local unfolding) along the protein sequence. These metrics allow us to locate areas with large % of lost contact time and high flexibility in urea (magenta), while orange and yellow regions illustrate large values of CC_UW_, meaning a remarkable preference to contact urea. For more examples see Suppl. Figure S6-B.

As noted above, residues that are very mobile in urea are also highly mobile in water at high temperature (see Suppl. Figure S4-A). Furthermore (see Suppl. Figure S4-C), with the exception of C22 simulations, there was a slight but significant (r>0.2; p-value:<2.2 10^−16^) correlation between oscillating residues in urea simulations and in native simulations (water at 300K). Interestingly, long-residence urea molecules were typically bound to rigid regions of the protein adjacent to mobile residues, i.e. they are located at putative hinge-points at the interface between the more rigid core of the protein and flexible loops or tails (see examples in Figure 6C and Suppl. Figure S6-C). The presence of sticky urea in these regions is expected to have a major role in guiding unfolding (see below).

## Discussion

MD simulations with additive potentials and explicit solvent have become very popular to explore chemical unfolding of protein. There is little doubt that the use of the technique has produced sizeable advances in the field, but we cannot ignore some potential caveats in the beginning of this discussion. First, for computational reasons we (and most authors in the field) are using classical non-polarizable force-fields, which might not be accurate enough to deal with a complex process such as unfolding. Previous studies [4]–[5], [7]–[15], [25] have however demonstrated that urea/water/protein effective parameters are able to reproduce a variety of experimental observables, such as mass densities and radial distribution functions of urea/water solutions derived from neutron scattering experiments [25], the experimental water/urea transfer free energies of tripeptides [10], and the urea density around unfolded proteins found by vapor pressure osmometry measures [4], [8], [10], [13], [15], [24]. Furthermore, our recent work [15] has demonstrated that unbiased MD simulations in 8M urea reproduce very accurately the unfolded ensemble as determined from a variety of spectroscopic techniques (including SAXS and NMR) under the same conditions. Thus, despite their simplicity current force-fields reproduce reasonably well urea/water/protein mixtures. We should remember that since we are exploring a microsecond-long process, no direct experimental data is available for comparison and accordingly caution is required. This move us to use a consensus approach, running the simulations with different force-fields to extract those results that seem robust to force-field changes.

A second reason of concern is related to the stochastic nature of unfolding, where individual trajectories can show different degree of unfolding [13]. Again, by comparing different trajectories we tried to define robust findings, but we cannot ignore that the experimental result is the averaging a near-Avogadro number of trajectories. A third reason of concern, common to many experimental studies, is the generality of the results, i.e. how general are the results obtained with a few model proteins. To convince ourselves on the general validity of our results we repeated the unfolding studies for a large number of proteins representative of all prevalent folds. Despite the obvious caveat of any theoretical study, this approach provided a picture of unprecedented, to our knowledge, completeness and robustness of the early stages of urea unfolding.

Under our simulation conditions (8M urea at T = 368 K), we detected clear signals of unfolding in the microsecond range, but progress in denaturation was smaller than that reported for model proteins of reduced stability [13]. Overall, the advance in unfolding of proteins at T = 368 after 1 µsec of MD is not dependent on the fold, nor secondary structure composition, and was similar for proteins with and without disulphide bridges in the native form. No dramatic differences in the advance of unfolding were found between water and urea simulations performed at the same temperature; however, unfolding paths in the presence or absence of urea differed, since the partially unfolded structures sampled in hot water maintained the hydrophobic residues hidden in the core of the protein, while such residues were more accessible in presence of urea.

Urea solutions are more viscous than pure water, which in our simulation reduced high-frequency movements in the protein, generating an unexpected slow down of the atomic-motions. Urea residence times around protein residues were large, especially when urea molecules diffuse close to the hydrophobic core or to the interface between rigid and thermally mobile regions (hinge points). We consider that the sticky nature of urea and its preferential placement at hinge points is crucial for unfolding, since it favors the rapid trapping of residues that become exposed as a consequence of stochastic thermal motions. The stabilizing effect of urea on exposed residues slowly biases the trajectory towards the unfolded state, by decreasing the chances of microscopic refolding [12], [26]. The effect of stabilization of exposed residues is especially productive in terms of unfolding when residues are apolar, since in this case urea (but not water) traps very efficiently the residue, increasing the accessibility of other apolar residues in the vicinity. The ensuing greater recruitment of urea in the region leads to a cooperative effect resulting in the acceleration of protein unfolding. Our data shows that, similar to the unfolded state [13], [15], it is the van der Waals interactions that drive the accumulation of urea on the surface of the folded protein. However, the role of H-bonding cannot be dismissed, as these bonds are crucial for the stabilization of long-living urea interactions near hinge points, which in turn are required to bias intrinsic protein dynamics towards unfolding. Clearly, “direct” effects not only are the main factors responsible for the urea-mediated stabilization of the unfolded state [15], but are also relevant in guiding the first steps of urea unfolding.

Microscopic unfolding events are related to stochastic thermal motions, which are in principle similar to those that occur spontaneously in water at room temperature. However, urea is not a mere passive spectator that simply stabilizes the small percentage of unfolded protein coexisting within the native ensemble and leading to a displacement in the folded←→unfolded equilibrium towards the denatured state. On the contrary, urea has a dual function: i) it takes advantage of microscopic unfolding events, decreasing their chances of refolding, and favoring further unfolding [12], [26]; and ii) among these microscopic unfolding events it selects and stabilizes microstates with exposed hydrophobic regions [4] (see Suppl. Figure S7). These effects lead to a slow divergence in the temperature-unfolding pathways in water and urea, and, as shown for ubiquitin [15], to distinct unfolded states. Consequently, concepts such as folded and unfolded states or folding and unfolding pathways need to be revisited and reformulated considering the nature of the denaturant used.

## Methods

### Selected proteins

As model structures for the main protein-folds we used the same structures selected in our previous work in reference 20. We first explored the early stages of urea unfolding using three ultra-representative proteins for the most populated fold in the three main classes in the SCOP database (*all-α* 1OPC, *all-β* 1CQY and *α/β* 1KTE; [19], [20]). Once the simulation protocols had been validated with these proteins, the study was extended to a larger set, consisting of 30 structures (110 residues on average) representative of the most populated protein folds ([20], [27] and Suppl. Dataset S1)

### Simulation set-up

All starting structures were taken from the Protein Data Bank (PDB; [28]) and processed using our standard procedure implemented in the MDWeb server [29]: experimental structures were titrated to define the major ionic state at neutral pH, neutralized by ions (sodium and chloride), minimized for 1000 steps, heated up to the final temperature, and solvated using a 8M urea/water octahedron box with a spacing distance of 15 Å around the system. The box was previously equilibrated in a Monte Carlo simulation using the BOSS program [30]. The water model was taken from Jorgensen's TIP3P [31], while ion and urea force-field parameters were those considered as the default of each force-field. Urea parameters from Smith et al. [32] were used for OPLS and P99SBILDN simulations, the same charges but scaled according to the amber force-field were used in PARM 99, while Nilsson's parameters were used in the CHARMM 22 force field [33]. Systems were then pre-equilibrated for 0.5 ns with parm99-AMBER force field in keeping the backbone restrained by intra-molecular harmonic potentials and then equilibrated (0.5 ns) in each force field parameters removing backbone constraints.

### Simulation details

For the small set of ultra-representative proteins, three sets of simulations corresponding to water at room temperature (T = 300 K), hot water (T = 368 K), and urea at high temperature (T = 368 K) were carried out. For each condition, we performed 1 µsec simulations using four force-fields: three general purpose ones (OPLSAA -ON2- [34]; CHARMM -C22- [35]; AMBER99 -P99- [36]), and a last-generation force-field able to accurately reproduce folded proteins (P99SBILDN, [37]). For the extended set of 30 proteins, control simulations in water were limited to 0.1 µsec at room temperature, while the 8M urea simulations were performed, as above, for 1 µsec at T = 368 K. Simulations for the extended set of proteins were carried out using ON2, C22 and P99. All simulations were performed using periodic boundary conditions and particle Mesh Ewald [38] corrections for the representation of long-range electrostatic effects using a 1.0 Å grid spacing and a 9 Å cutoff. All trajectories were collected with the NAMD2 [39] program. Integration of equations of motions was performed every 2 fs after removing vibrations of bonds involving hydrogen atoms using SHAKE/RATTLE algorithm [40], [41]. All simulations were carried out in the isothermal (T = 300 or 368 K)/isobaric ensemble (P = 1 atm) using the Langevin thermostat and barostats [42], [43]. The trajectories were analyzed using VMD [44] and the MdWeb server [29], as well as Flexserver which can be accessed at: http://mmb.pcb.ub.es/FlexServ/ (see also Suppl. Text S1 for a detailed explanation of the metrics).

## Supporting Information

Dataset S1 **List of the structures selected to represent the 30 most populated folds according to SCOP, CATH, Dali and Dagget's databases** [**20**] **.** When available, we included the denaturation midpoint, as measurement of protein intrinsic stability.
(XLS)

Figure S1 **Structural descriptors for the ultra representative proteins.** Structural descriptors (and associated standard deviations) for the 3 ultra representative proteins along the first and last 10 ns of the simulated time (1 microsecond) in water at 300K. The red line reports values for the starting conformation. Error bars mark the standard deviation.
(TIF)

Figure S2 **Root mean square deviations for the ultra representative proteins.** **A**) RMSd evolution and **B**) distribution among 1 microsecond for the 3 ultra representative proteins in the three environments: water at 300K, urea 8M at 368K and water at 368K. Each color identifies a force-field: red for C22, violet for ON2, blue for P99 and green for P99SBILDN.
(TIFF)

Figure S3 **Evolution of solvent accessible areas for the ultra representative proteins.** Correlation between solvent accessible surface area (ΔSASA) of polar (left side) and apolar residues (right) and the global structure index. For each force-field, values for the three ultra-representative proteins: 1KTE (green), 1CQY (red) and 1OPC (blue) are reported. **Δ**SASA is defined as the difference to the average values of the corresponding control simulations, the global structure index is used to follow the progress in the unfolding process (from 1 - fully native folded protein - towards 0).
(TIFF)

Figure S4 **Comparison between unfolding in hot water and urea for the ultra representative proteins.** **A**) Correlation between the percentages of lost contact time for each residue in urea and in hot water (r = 0.701; p-value<2.2 10^−16^). The percentage of lost contact time is calculated as contact time lost during 1 microsecond (using water simulation at 300 K as a reference) **B**) Average RMSd measured in different time windows (time lag), from 2 ns up to 200 ns, in hot water (blue) and urea (green). Reference structure for RMSd calculations is always the first frame in the window, which means tha this metrics gives an estimate of the short time scale oscillations of the protein **C**) Force-field dependent distribution of average opening times(temporal unfold – see Suppl. Text S1) in urea (green) and hot water (orange) during the first 100 ns of simulations for the three ultra-representative proteins. **D**) Correlation between the root mean square fluctuation (RMSF) of the residues between simulations in urea (368K) and water (300K). P-value is always smaller than 2.2 10^−16^.
(TIFF)

Figure S5 **Solvent features in urea unfolding simulations.** **A**) Average ratio water/urea molecules in the first solvation shell of the 30 representative proteins in urea (values for every force-field are presented using normal color code). Average values and relative standard deviations are calculated in the last 10 ns of the simulation. To facilitate discussion proteins are grouped according to the SCOP classification, the group average is reported as AV while the symbol * marks proteins with disulfide bonds. Error bars mark the standard deviation. **B**) Distribution of Van der Waals and electrostatic energies for urea and water in the first solvation shell and in the bulk. **C**) Urea and water mean square displacement in different time windows (tau) among the last 10 ns of the trajectories. The diffusion coefficient is calculated using the Einstein equation, more details in Suppl. Text S1.
(PDF)

Figure S6 **Examples of urea contacts during protein unfolding.** **A**) Examples of urea-protein contacts along simulation time (µsec). Each dot in the plot defines a contact between that particular urea molecule and a residue in the protein. Examples of urea molecules trapped in the protein core are shown. **B**) Variation along the sequence of the residue RMSF (measure for the flexibility), the contact coefficient CC_UW_ (measure for the preference of protein to contact urea vs. water) and the % of lost contact time (Lost; a measure for the unfolding). The three examples are randomly chosen among the 30 simulations; results are shown for all the three force-field. The RMSF for each residue is calculated in water (green) and in urea (red) while the B-factors (appropriately scaled to maintain same units as RMSF) are from the PDB structure (blue). Residues that are part of protein core are marked in yellow along the x-axes. The color scale for CC_UW_ along the protein sequence ranges from blue (low preference for urea) to orange (large preference for urea). Areas of high urea preference are mostly located in rigid regions flanking highly flexible segments. The % of lost time is calculated as the average percentage of lost time from all the native contacts that each residue forms. The color scale ranges from blue (low unfolding) to magenta (large unfolding).
(TIFF)

Figure S7 **A scheme to illustrate the action of urea on micro-folding events.** Two residues exposed due to local unfolding oscillation - that quickly re-collapse in water - can remain exposed for longer time in presence of urea. Urea, that has a greater ability than water to form dispersion interactions, can stabilize parts of the protein that are usually hidden from the solvent, such as hydrophobic residues, and that can become exposed during these unfolding oscillation. The summation of many of these events moves the equilibrium towards the unfolding state of a protein.
(TIF)

Table S1 **Comparison of structural descriptors for 3 ultra-representative proteins in the periods (10–100 ns) and (910–1000 ns).**
(DOCX)

Table S2 **Comparison of structural descriptors for 3 ultra-representative proteins in the period (990–1000 ns) calculated in hotwater (HW) and urea (U) and their difference with water (W) among the same period.** Values are displayed as mean(standard deviation).
(DOCX)

Table S3 **Comparison of % secondary structure for 3 ultra-representative proteins in the period (990–1000 ns) calculated in hotwater (HW), urea (U) and water (W).**
(DOCX)

Table S4 **Hydrogen bond interactions of urea/water with proteins during the last 10 ns of trajectories for different force-fields.** Life-time refers always to the 10 ns window analyzed.
(DOCX)

Text S1 **Methods.** Description of the analysis performed.
(DOCX)
