# Supplementary figures and images for: Exploring Early Stages of the Chemical Unfolding of Proteins at the Proteome Scale
Source: PLoS Comput Biol. 2013 Dec 12;9(12):e1003393. doi: 10.1371/journal.pcbi.1003393 (PMC3861036; doi:10.1371/journal.pcbi.1003393)

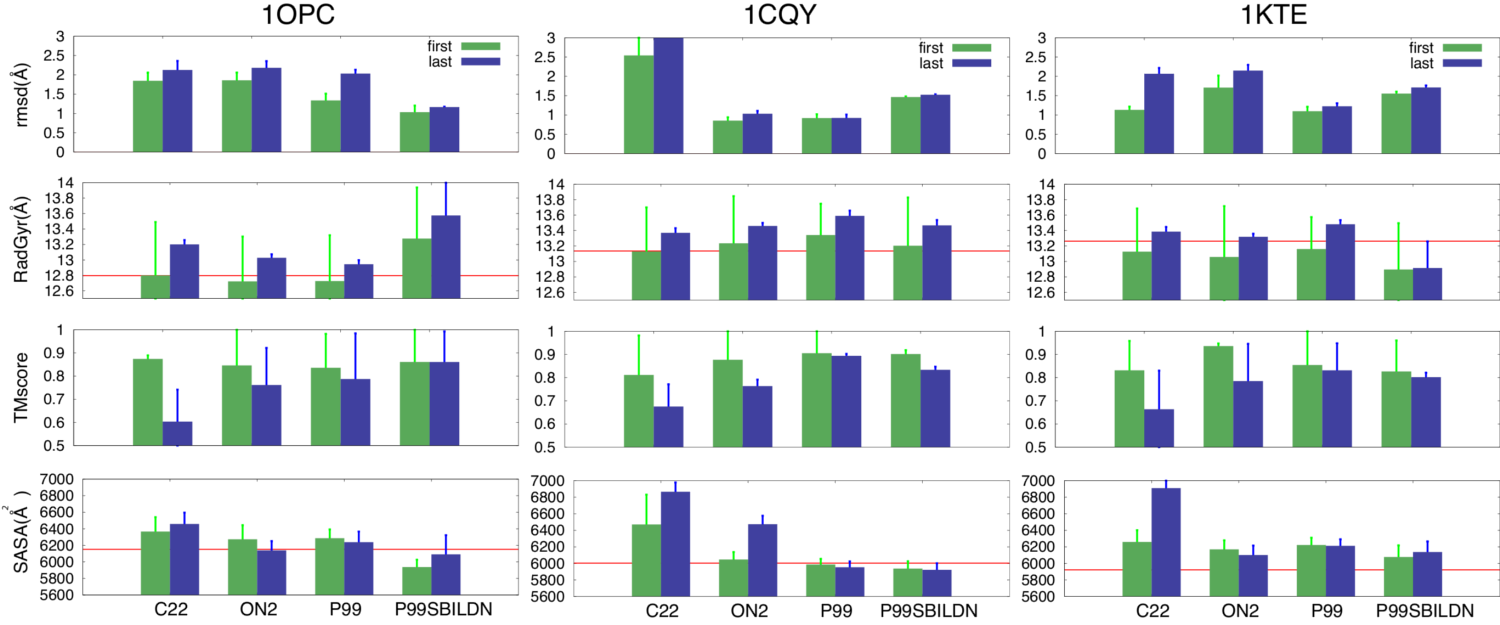

Supplement: Figure S1 — Structural descriptors for the ultra representative proteins. Structural descriptors (and associated standard deviations) for the 3 ultra representative proteins along the first and last 10 ns of the simulated time (1 microsecond) in water at 300K. The red line reports values for the starting conformation. Error bars mark the standard deviation. (TIF) [file pcbi.1003393.s002.tif]

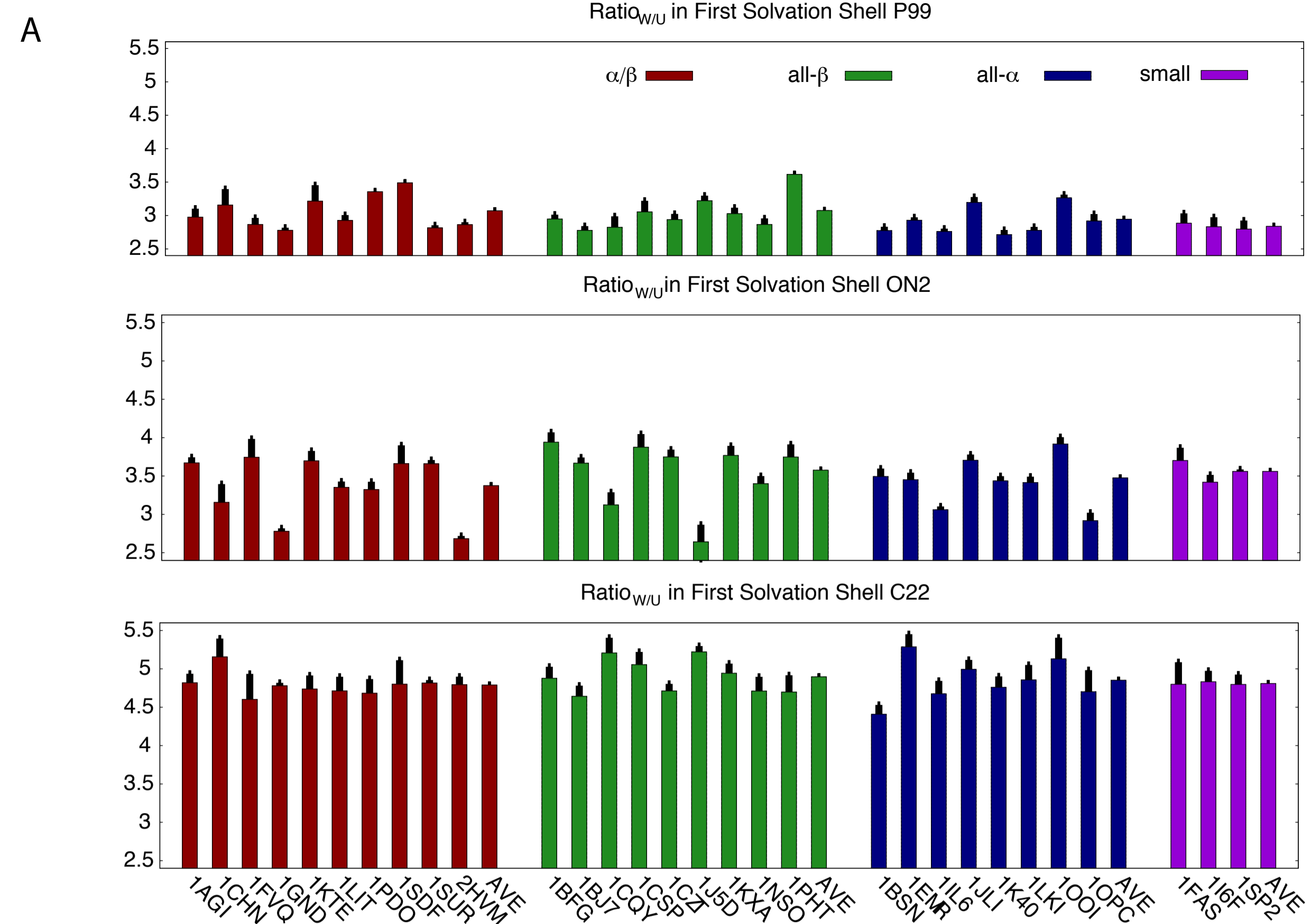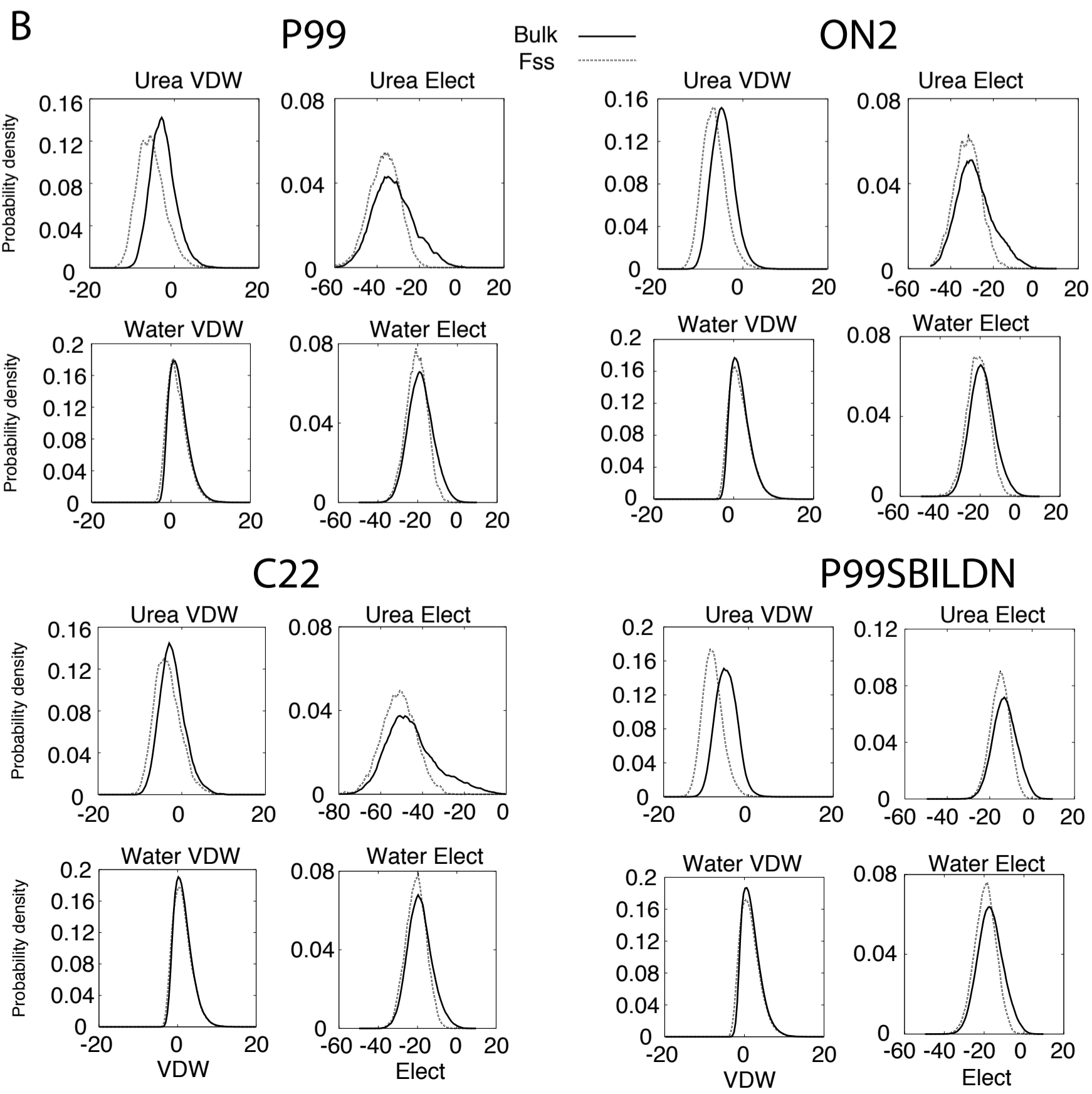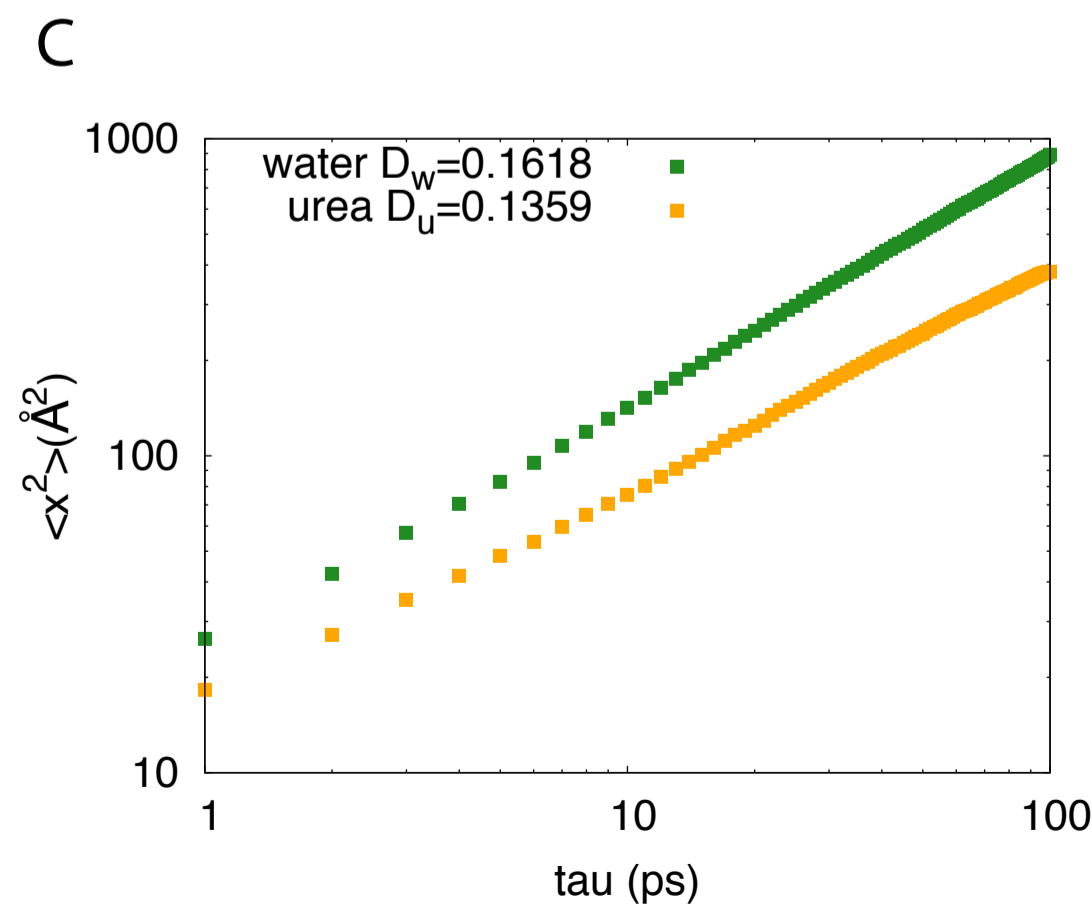

Supplement: Figure S5 — Solvent features in urea unfolding simulations. A) Average ratio water/urea molecules in the first solvation shell of the 30 representative proteins in urea (values for every force-field are presented using normal color code). Average values and relative standard deviations are calculated in the last 10 ns of the simulation. To facilitate discussion proteins are grouped according to the SCOP classification, the group average is reported as AV while the symbol * marks proteins with disulfide bonds. Error bars mark the standard deviation. B) Distribution of Van der Waals and electrostatic energies for urea and water in the first solvation shell and in the bulk. C) Urea and water mean square displacement in different time windows (tau) among the last 10 ns of the trajectories. The diffusion coefficient is calculated using the Einstein equation, more details in Suppl. Text S1. (PDF) [file pcbi.1003393.s006.pdf]

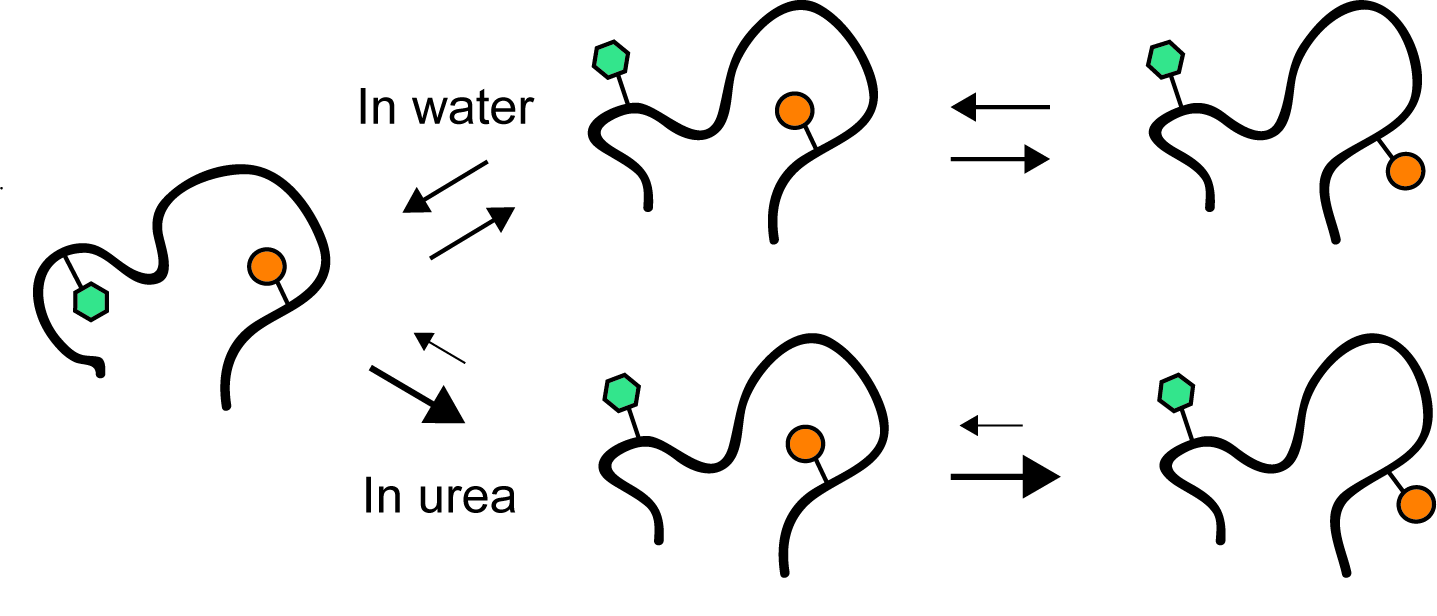

Supplement: Figure S7 — A scheme to illustrate the action of urea on micro-folding events. Two residues exposed due to local unfolding oscillation - that quickly re-collapse in water - can remain exposed for longer time in presence of urea. Urea, that has a greater ability than water to form dispersion interactions, can stabilize parts of the protein that are usually hidden from the solvent, such as hydrophobic residues, and that can become exposed during these unfolding oscillation. The summation of many of these events moves the equilibrium towards the unfolding state of a protein. (TIF) [file pcbi.1003393.s008.tif]
