# Supplementary material for: Exploring Early Stages of the Chemical Unfolding of Proteins at the Proteome Scale
Source: PLoS Comput Biol. 2013 Dec 12;9(12):e1003393. doi: 10.1371/journal.pcbi.1003393 (PMC3861036; doi:10.1371/journal.pcbi.1003393)
Supplement: Table S1 — Comparison of structural descriptors for 3 ultra-representative proteins in the periods (10–100 ns) and (910–1000 ns). (DOCX) [file pcbi.1003393.s009.docx]

**Table S1.** Comparison of structural descriptors for 3 ultra-representative proteins in the first part of the simulation time (10-100 ns) and in the last (910-1000 ns) and their difference (Δ(Last-First)). When possible values are displayed as mean(standard deviation) and the Z-score has been calculated of the Δ(Last-First) value related to the differences between non consequitive windows of 10 ns.

| Structural Descriptor |  | FIRST  (10-100ns) | LAST  (910-1000ns) | Δ(Last-First) | Z-score |
| --- | --- | --- | --- | --- | --- |
| Rmsd(Å) | C22  ON2  P99  P99* | 1.84(0.29)  1.47(0.20)  1.12(0.13)  1.35(0.08) | 2.54(0.19)  1.78(0.13)  1.39(0.09)  1.46(0.02) | 0.70(0.09)  0.31(0.06)  0.27(0.04)  0.11(0.05) | 1.34  0.77  0.63  0.34 |
| Tmscore | C22  ON2  P99  P99* | 0.84(0.10)  0.89(0.12)  0.87(0.16)  0.86(0.11) | 0.65(0.13)  0.77(0.12)  0.84(0.11)  0.83(0.06) | -0.19(0.03)  -0.12(0.00)  -0.03(-0.05)  -0.03(-0.05) | -1.31  0.78  0.56  0.46 |
| R_g_ (Å) | C22  ON2  P99  P99* | 13.02(0.61)  13.00(0.62)  13.08(0.47)  13.12(0.63) | 13.32(0.06)  13.27(0.04)  13.34(0.06)  13.32(0.28) | 0.30(-0.54)  0.26(-0.57)  0.26(-0.41)  0.19(-0.35) | 0.59  0.68  0.69  0.43 |
| SASA (Å^2^) | C22  ON2  P99  P99* | 6365(225)  6164(123)  6165(88)  5984(107) | 6744(147)  6237(110)  6134(93)  6050(147) | 378(-80)  74(-13)  -30(5)  66(40) | 1.23  0.89  0.23  0.65 |
| % Helix | C22  ON2  P99  P99* | 25.3  24.6  25.0  25.3 | 25.3  25.9  24.6  25.6 | 0.0  1.3  -0.4  0.3 |  |
| % Sheets | C22  ON2  P99  P99* | 27.9  29.7  29.9  30.1 | 24.3  28.9  27.8  30.0 | -3.6  -0.8  -2.1  -0.1 |  |
