# Supplementary material for: Exploring Early Stages of the Chemical Unfolding of Proteins at the Proteome Scale
Source: PLoS Comput Biol. 2013 Dec 12;9(12):e1003393. doi: 10.1371/journal.pcbi.1003393 (PMC3861036; doi:10.1371/journal.pcbi.1003393)
Supplement: Table S2 — Comparison of structural descriptors for 3 ultra-representative proteins in the period (990–1000 ns) calculated in hotwater (HW) and urea (U) and their difference with water (W) among the same period. Values are displayed as mean(standard deviation). (DOCX) [file pcbi.1003393.s010.docx]

**Table S2.** Comparison of structural descriptors for 3 ultra-representative proteins in the period (990-1000 ns) calculated in hotwater(HW) and urea (U) and their difference with water (W) among the same period. Values are displayed as mean(standard deviation).

|  | Hot Water(HW)  (990-1000ns) | Urea (U)  (990-1000ns) | Δ(HW-W) | Δ(U-W) |
| --- | --- | --- | --- | --- |
| Rmsd (Å) C22  ON2  P99  P99* | 7.35(1.25)  4.91(0.24)  7.49(0.51)  4.89(0.20) | 6.01(0.33)  5.11(0.19)  7.64(0.68)  7.74(0.32) | 4.68(1.10)  3.15(0.00)  6.13(0.44)  3.72(0.10) | 3.33(0.18)  3.36(-0.04)  6.28(0.61)  6.58(0.22) |
| Tmscore C22  ON2  P99  P99* | 0.40(0.06)  0.60(0.11)  0.45(0.06)  0.48(0.10) | 0.53(0.07)  0.60(0.02)  0.41(0.11)  0.58(0.05) | -0.24(-0.05)  -0.18(0.027)  -0.39(-0.01)  -0.39(-0.00) | -0.11(-0.03)  -0.18(-0.05)  -0.44(0.04)  -0.28(-0.05) |
| R_g_(Å) C22  ON2  P99  P99* | 13.96(0.31)  13.50(0.14)  13.67(0.24)  13.64(0.15) | 14.27(0.21)  13.91(0.09)  13.79(0.16)  14.93(0.28) | 0.37(0.20)  0.13(0.06)  0.35(0.18)  0.29(0.08) | 0.68(0.10)  0.55(0.02)  0.47(0.10)  1.58(0.20) |
| SASA(Å^2^) C22  ON2  P99  P99* | 7418(295)  6372(149)  6607(190)  6570(160) | 7302(176)  6822(125)  7007(204)  7517(210) | 691(152)  126(55)  516(124)  533(59) | 575(33)  576(31)  915(138)  1479(108) |
| S_2_ (%) C22  ON2  P99  P99* | 56.52(2.76)  58.10(2.28)  64.24(3.06)  74.59(1.41) | 66.57(2.53)  64.48(2.02)  51.34(1.24)  55.10(2.01) | -17.56(0.95)  -20.6(0.10)  -18.35(1.3)  -12.0(-0.34) | -7.52(0.72)  -14.22(-0.15)  -31.26(-0.52)  -31.50(0.24) |
| S_3_(%) C22  ON2  P99  P99* | 36.39(5.28)  58.83(6.57)  46.20(9.44)  69.43(5.16) | 51.95(8.6)  64.13(5.29)  32.66(5.14)  45.56(4.27) | -43.27(3.04)  -22.87(4.39)  -39.72(7.68)  -17.53(3.55) | -27.71(6.37)  -17.57(3.11)  -53.26(3.38)  -41.41(2.66) |
