# Supplementary material for: Exploring Early Stages of the Chemical Unfolding of Proteins at the Proteome Scale
Source: PLoS Comput Biol. 2013 Dec 12;9(12):e1003393. doi: 10.1371/journal.pcbi.1003393 (PMC3861036; doi:10.1371/journal.pcbi.1003393)
Supplement: Table S3 — Comparison of % secondary structure for 3 ultra-representative proteins in the period (990–1000 ns) calculated in hotwater (HW), urea (U) and water (W). (DOCX) [file pcbi.1003393.s011.docx]

**Table S3.** Comparison of % secondary structure for 3 ultra-representative proteins in the period (990-1000 ns) calculated in hotwater(HW), urea (U) and water (W).

|  | Hot Water(HW)  % alpha / beta | Urea (U)  % alpha / beta | Water(W)  % alpha / beta |
| --- | --- | --- | --- |
| 1KTE C22  ON2  P99  P99* | 25 / 10  14 / 22  34 / 12  28 / 20 | 37 / 12  26 / 17  24 / 9  11 / 9 | 41 / 17  41 / 16  39 / 17  39 / 20 |
| 1OPC C22  ON2  P99  P99* | 26 / 4.0  28 / 18  32 / 16  28 / 14 | 32 / 11  27 / 18  26 / 5  25 / 10 | 34 / 18  31 / 23  35 / 19  33 /23 |
| 1CQY C22  ON2  P99  P99* | 1 / 22  0 / 46  9 / 31  2 / 46 | 0 / 30  0 / 39  7 / 34  2 / 46 | 0 / 52  2 /56  1 /48  3 / 48 |
