# Supplementary material for: Exploring Early Stages of the Chemical Unfolding of Proteins at the Proteome Scale
Source: PLoS Comput Biol. 2013 Dec 12;9(12):e1003393. doi: 10.1371/journal.pcbi.1003393 (PMC3861036; doi:10.1371/journal.pcbi.1003393)
Supplement: Table S4 — Hydrogen bond interactions of urea/water with proteins during the last 10 ns of trajectories for different force-fields. Life-time refers always to the 10 ns window analyzed. (DOCX) [file pcbi.1003393.s012.docx]

**Table S4:** Hydrogen bond interactions of urea / water with proteins during the last 10 ns of trajectories for different force-fields. Life-time refers always to the 10 ns window analyzed.

**a) OPLS**

| **H- bonds*:** | **Urea / Water as H-donor** | | **Urea /Water as H-acceptor** | |  |
| --- | --- | --- | --- | --- | --- |
| % of total | 64 / 62 | | 36 / 38 | |  |
| **H-bonds with protein:** | **BackBone** | **SideChains** | **BackBone** | **SideChains** | |
| % of total | 64 / 46 | 36 / 54 | 70 / 72 | 30 / 28 | |
| Lifetime % | 0.69 / 0.58 | 1.08 / 0.80 | 4.25 / 1.01 | 4.18 / 3.79 | |

**b) CHARMM**

| **H- bonds*:** | **Urea / Water as H-donor** | | **Urea / Water as H-acceptor** | |  |
| --- | --- | --- | --- | --- | --- |
| % of total | 66 / 60 | | 34 / 30 | |  |
| **H-bonds with protein:** | **BackBone** | **SideChains** | **BackBone** | **SideChains** | |
| % of total | 53 / 55 | 47 / 45 | 64 / 69 | 36 / 31 | |
| Lifetime % | 0.84 / 0.63 | 1.28 / 0.72 | 2.75 / 0.56 | 4.71 / 2.29 | |

**c) PARM 99**

| **H- bonds*:** | **Urea / Water as H-donor** | | **Urea / Water as H-acceptor** | |  |
| --- | --- | --- | --- | --- | --- |
| % of total | 69 / 65 | | 31 / 35 | |  |
| **H-bonds with protein:** | **BackBone** | **SideChains** | **BackBone** | **SideChains** | |
| % of total | 55 / 44 | 45 / 56 | 67 / 69 | 33 / 31 | |
| Lifetime % | 0.73 / 0.66 | 0.94/ 0.68 | 2.67 / 0.54 | 3.94 / 2.09 | |
