# Supplementary material for: Exploring Early Stages of the Chemical Unfolding of Proteins at the Proteome Scale
Source: PLoS Comput Biol. 2013 Dec 12;9(12):e1003393. doi: 10.1371/journal.pcbi.1003393 (PMC3861036; doi:10.1371/journal.pcbi.1003393)
Supplement: Text S1 — Methods. Description of the analysis performed. (DOCX) [file pcbi.1003393.s013.docx]

**Supplementary Text S1 -** Methods

**Analysis**. Trajectories were analyzed using a variety of metrics. Protein structural descriptor include Root Mean Square Deviation (RMSD), TMscore, Radius of Gyration (RadGyr), Secondary Structure (SS - evaluated using STRIDE [1]), Solvent Accessible Surface Area (SASA - evaluated using NACESS [2]). The average RMSD was measured in different time windows, with a time lag from 2 ns up to 200 ns, in water and urea at 368K. and always using as reference structure the first frame in the window.

To describe the unfolding , we calculated the change of protein features taking as the reference the native state described by the control simulation at 300K in water. Therefore the native contacts (tertiary structure) and native secondary structure were calculated as those occurring for more than 80% of the time in the control simulation, while the protein core was considered formed by residues with an average SASA and standard deviation lower then 10 Å^2^ in the control simulation.

For the trajectories in urea and water at 368K we calculated the secondary stucture index “S2” as the existing fraction of native secondary structure (see above) in each frame, and the tertiary structure index “S3” as the existing fraction of native contacts in each frame. Residues were considered to be in contact when their interesidue distance was shorter than 3.5 Å [3]. The global structure index [4] was defined as the sum of S2 and S3.

Regarding the stability of intra-protein contats, we considered as lost contacts those with a reduced contact time in urea or water at 368K compared to water simulation at 300 K (reduction for more than 30% of the simulated time). The % of lost time for a residue was calculated as the average percentage of lost contact time for all the native contacts involving that residue, during 1 microsecond in urea or water at 368K and using water simulation at 300K as a reference. The flexibility of the contacts and the average opening time was calculated for each native contact at each snapshot in the first 100ns of hot water and urea simulations. This first part of the simulation contains the largest number of comparable contacts (see below), in later stages most of the contacts are generally unstable at least in one of the environments and therefore a comparison would be uninformative. A contact was considered “open” if the minimum distance between heavy atoms was larger than 5 Å and “closed” if the distance was smaller than 4 Å. In the moonlight zone ( between 4 and 5 Å) the contact assumed the state of the previous frame, avoiding ambiguous classifications. We focused the analysis on comparable contacts that are still preserved in both urea and water at 368K ( difference in contact time is less than 20% compared to water at 300 K, in both simulations). Contacts that are completely lost or fully maintained in at least one of the two environments were removed because they are uninformative regarding to changes in flexibility. We calculated the rmsf_SC_ as the rmsf for a single sidechain after an alignment based only on the backbone of the same residue- thus the metric is only dependent on the local motion. The difference of rmsf_SC_ between water and urea at 368K was used to evaluate the change in sidechain dynamics. We excluded differences smaller than 0.5 to avoid the comparison of residues with similar flexibility. Therefore the analysis was performed on values for ∆ rmsf_SC_ (rmsf_SC_ in water – rmsf_SC_ in urea) larger than 0.5 or smaller than -0.5.

Solvent features evaluated here include water/urea ratio in first solvatation shell (FSS; solvent molecules within 5 Å of the protein) and in the bulk (solvent molecules with a distance to the protein larger than 6Å). More detailed analysis were perfomed using the contact coefficient CC_UW_ metric. CC_UW_ is the ratio for each aminoacid between contacts with urea and with water molecules normalized with the total numbers of urea and water atoms [3]; a contact is formed when at least two heavy atoms are closer than 3.5 Å. The residence time for urea and water molecules during 1 microsecond trajectory was calculated as the time each solvent molecule is in contact (see previous definition of contact) with the same residues without any interruptions. Urea and water mean square displacements were calculated in different time windows (tau) among the last 10 ns of the trajectories. We used the Einstein equation [5] to calculate the diffusion coefficient (D) from the slope of the fitting line. Since the Einstein relation is valid as time approaches infinity, we used only the last half of values for the fitting.

Solvent-protein hydrogen bonds were annotated with a heavy atom cutoff distance of 3.5 Å and a donor-hydrogen-acceptor angle greater than 120 degree. Stable H-bonds were defined as those detected for more than 5% of the analyzed time. Interaction energies for urea and water in the FSS and bulk were computed following Hua et al. [6] using a 13.0Å spherical cutoff .

All the analyses were perform with MDWEB [7], VMD [8] , Ptraj [9] and in house software, while statistical analysis were perfomed with R [10].

**Supplementary Bibliography**

1. Heinig, M., Frishman, D. (2004). STRIDE: a Web server for secondary structure assignment from known atomic coordinates of proteins. Nucl. Acids Res. , 32, W500-2.

2. Hubbard,S.J.& Thornton, J.M. (1993), 'NACCESS', Computer Program, Department of Biochemistry and Molecular Biology, University College London."

3. Stumpe M. C. and Grubmüller H. (2007) Interaction of Urea with Amino Acids - Implications for Urea-Induced Protein Denaturation. *J.Am.Chem.Soc.*129(51):16126-31

4. Simms A.M., Toofanny R.D., Kehl C., Benson N.C., and Daggett V. (2008) Dynameomics: design of a computational lab workflow and scientific data repository for protein simulations. Protein Engineering Design & Selection 21: 369-377.

5. Allen, M. P., Tildesley, D. J. (1987) Computer Simulations of Liquids. Oxford: Oxford Science Publications.

6. Hua L,; Zhou R,; Thirumalai D,; Berne BJ. (2008) Urea denaturation by stronger dispersion interactions with proteins than water implies a 2-stage unfolding. [Proc Natl Acad Sci U S A.](http://www.ncbi.nlm.nih.gov/pubmed/18957546) 2008 Nov 4;105(44):16928-33

7. Hospital A, Andrio P, Fenollosa C, Cicin-Sain D, Orozco M, Gelpí JL. (2012) [MDWeb and MDMoby: an integrated web-based platform for molecular dynamics simulations.](http://bioinformatics.oxfordjournals.org/content/28/9/1278) Bioinformatics 28(9):1278-9.

8. Humphrey, W., Dalke, A. and Schulten, K., ``VMD - Visual Molecular Dynamics'' *J. Molec. Graphics* **1996**, *14.1*, 33-38.

9. D.A. Case, T.A. Darden, T.E. Cheatham, III, C.L. Simmerling, J. Wang, R.E. Duke, R. Luo, R.C. Walker, W. Zhang, K.M. Merz, B. Roberts, S. Hayik, A. Roitberg, G. Seabra, J. Swails, A.W. Goetz, I. Kolossváry, K.F. Wong, F. Paesani, J. Vanicek, R.M. Wolf, J. Liu, X. Wu, S.R. Brozell, T. Steinbrecher, H. Gohlke, Q. Cai, X. Ye, J. Wang, M.-J. Hsieh, G. Cui, D.R. Roe, D.H. Mathews, M.G. Seetin, R. Salomon-Ferrer, C. Sagui, V. Babin, T. Luchko, S. Gusarov, A. Kovalenko, and P.A. Kollman (2012), *AMBER 12*, University of California, San Francisco.

10. R Development Core Team (2008). R: A language and environment for statistical computing. R Foundation for Statistical Computing, Vienna, Austria. ISBN 3-900051-07-0, URL [http://www.R-project.org.](http://www.R-project.org./)
